# The next paradigm in bioinformatics: a review of multi-agent systems and foundational models for end-to-end scientific discovery

**DOI:** 10.1093/bib/bbag245

**Published:** 2026-05-20

**Authors:** Francesco Branda, Mohamed M Ahmed, Massimo Ciccozzi, Pietro Hiram Guzzi, Fabio Scarpa

**Affiliations:** Unit of Medical Statistics and Molecular Epidemiology, Università Campus Bio-Medico di Roma, Via Álvaro del Portillo, 21, 00128 Rome, Italy; Faculty of Medicine and Health Sciences, SIMAD University, Mogadishu 252, Somalia; Unit of Medical Statistics and Molecular Epidemiology, Università Campus Bio-Medico di Roma, Via Álvaro del Portillo, 21, 00128 Rome, Italy; Department of Surgical and Medical Sciences, Magna Graecia University of Catanzaro, Viale Europa, 88100 Catanzaro, Italy; Department of Biomedical Sciences, University of Sassari, Viale San Pietro, 07100 Sassari, Italy

**Keywords:** agentic AI, foundation models, multi-agent systems, drug discovery, personalized medicine, retrieval-augmented generation (RAG)

## Abstract

Bioinformatics is entering a new phase characterized by the integration of universal biological models and multi-agent systems to enable end-to-end scientific discoveries. This review argues that the next paradigm shift will go beyond traditional predictive models and generative artificial intelligence (AI) toward agentic AI: systems capable of planning, acting through tools, reflecting on results, and iterating until a goal is achieved. We first examine recent foundational models that produce transferable representations across omic modalities, such as scGPT, Nicheformer, and EpiAgent, and discuss their architectural choices, training regimes, and interpretability constraints. We then analyze biomedical agent frameworks through their main components (planning, action, reflection, and memory), highlighting representative systems such as ClinicalAgent and Biomni that operationalize these ideas in controlled environments. Next, we focus on hypothesis validation mechanisms, including retrieval-augmented generation for evidence grounding, sequential statistical testing, and benchmarking methodologies designed to quantify robustness and reproducibility. Finally, we summarize emerging applications in drug discovery and personalized medicine, from molecular literature analysis and protocol automation to drug repurposing for rare diseases and closed-loop synthesis. We conclude by outlining the main challenges ahead, namely hallucinations, interpretability, systemic biases, integration with clinical infrastructures, and regulatory and ethical requirements, and propose a roadmap for the development of scientific agents that are not only high-performing but also reliable, verifiable, and implementable in real biomedical contexts.

## Introduction

Bioinformatics is entering a historic phase in which the fundamental question is no longer whether machine learning can classify variants or predict phenotypes, but rather whether artificial intelligence (AI) systems are capable of performing substantial portions of the scientific process in an end-to-end, auditable, and reproducible manner. Over the past 15 years, the field has been shaped by 3 partially overlapping waves, each characterized by a different level of abstraction and computational autonomy.

The first wave saw the emergence of deep learning as the dominant paradigm for analyzing high-dimensional biological data. Deep neural architectures have progressively replaced pipelines based on manually engineered features, demonstrating superior performance in tasks such as genomic variant classification, protein function prediction, regulatory element annotation, and phenotypic stratification [1–3]. In this regime, the scientific problem was formalized as a well-defined “input → output” mapping: a genomic sequence, an expression profile, or an omic count matrix constituted the input; the output was a label, a probability, or a continuous score. The goal was predictive accuracy, measured on static benchmarks. This phase consolidated robust methodological infrastructures but maintained a clear separation between statistical modeling and the broader scientific process.

In parallel with these developments, AI has also enabled major advances in complementary bioinformatics domains that underpin modern computational biology pipelines. In sequence analysis and representation learning, scalable computational frameworks and deep learning models have significantly improved the analysis of large genomic and transcriptomic datasets. Recent large-scale genome alignment approaches enable efficient comparative analysis across thousands to millions of sequences, facilitating evolutionary and epidemiological investigations [4]. Similarly, deep-learning architectures integrating sequence and structural information have substantially improved ribonucleic acid (RNA) secondary structure prediction and functional inference, demonstrating the potential of representation learning in modeling complex molecular structures [5].

The second wave was driven by the emergence of attention mechanisms and sequence-to-sequence models [6], culminating in foundation models and large language models [7]. In biology, this led to a shift in perspective: no longer just supervised prediction, but large-scale self-supervised learning and the construction of transferable representations. Language models for proteins have shown that structural and functional principles emerge spontaneously from training on millions of unannotated sequences [8], while systems such as AlphaFold have demonstrated that the integration of deep learning and physical-structural constraints can achieve accuracy comparable to that of experiments in predicting three-dimensional structure [9]. In parallel, foundation models for single-cell omics have begun to produce universal embeddings of cells and genes that are robust to batch effects and technical variability and transferable across tissues, conditions, and platforms [10, 11]. In this context, the model becomes an epistemic infrastructure: a shared latent space that encodes biological regularities and enables systematic reuse of knowledge.

More broadly, recent AI-driven approaches increasingly integrate heterogeneous biomedical data modalities. Multi-omic integration frameworks combine genomic, transcriptomic, epigenomic, and clinical information to improve disease diagnosis and identify candidate therapeutic targets, leveraging multimodal learning strategies to capture cross-modal biological associations [12, 13]. In clinical and translational contexts, machine learning techniques have also transformed the analysis of medical imaging and biomedical signals, supporting automated interpretation and decision support across radiological and pathological modalities [14]. In parallel, hybrid computational approaches combining biological network with machine learning models have demonstrated promise in identifying drug–target relationships and prioritizing therapeutic candidates in drug discovery pipelines [15]. Furthermore, advances in spatial omics technologies have stimulated the development of computational frameworks capable of modeling spatially resolved gene expression, reconstructing tissue microenvironments, and identifying spatially variable genes across complex tissues [16].

A third wave is now emerging, defined by agentic AI. Here, the model is not only a predictor or generator, but also a system capable of planning, using external tools, reflecting on the outcomes of its actions, and iterating until an explicit goal is achieved [17]. The typical architecture integrates four fundamental components: (i) a planning module that decomposes a complex goal into subtasks; (ii) tool use interfaces to query databases, execute bioinformatics pipelines, or control experimental automations; (iii) short- and long-term memory, often implemented through retrieval-augmented generation (RAG), to maintain context and documentary grounding [18]; and (iv) reflection and self-assessment mechanisms to detect inconsistencies, estimate uncertainty, and correct errors.

In biomedicine, this paradigm is particularly promising because discovery workflows are inherently modular and multistage: literature review, multimodal integration of genomic, epigenomic, and transcriptomic data, generation of mechanistic hypotheses, experimental design, statistical analysis, and validation. Each stage introduces degrees of analytical freedom and potential sources of bias, contributing to the reproducibility crisis documented in the scientific literature [19]. Multi-agent systems coordinated collections of agents with specialized roles (e.g. “literature analyst,” “pipeline executor,” “statistical validator,” “experimental planner”), offer a natural abstraction for orchestrating such workflows while maintaining a detailed audit trail: sequence of evidence retrieved, analysis parameters, intermediate outputs, and decision rationales.

A scientific agent could, for example, automatically identify relevant studies on a molecular target, integrate public datasets, perform standardized differential analyses, propose a candidate regulatory network, and design a validation experiment under explicit constraints of cost, time, and sample availability. In clinical or drug discovery contexts, however, such systems must meet more stringent requirements than traditional benchmarks: stability of inferences with respect to small perturbations, calibration of uncertainty, prevention of data leakage, and systematic grounding on verifiable evidence.

In this perspective, the next paradigm of bioinformatics will be defined by the integration of three pillars: (i) universal biological foundation models capable of learning representations that are transferable between omic modalities and species; (ii) multi-agent architectures for reasoning and instrumental action; and (iii) automated validation systems incorporating sequential statistical testing, pipeline auditing, and benchmarks oriented toward robustness and reproducibility. The goal is not only to maximize predictive accuracy, but to build epistemically reliable systems: capable of documenting their inferential path, making assumptions and limitations explicit, and operating consistently in real, regulated, high-impact environments.

### Limitations of single-pillar approaches and the necessity of integration

The three pillars proposed in this work, i.e. universal foundation models (Pillar I), multi-agent architectures (Pillar II), and automated validation systems (Pillar III), are not merely complementary components that independently improve upon the current state of the art. Rather, the principal motivation for the integrated paradigm arises from the characteristic failure modes that emerge when any subset of these pillars is deployed in isolation. Making these limitations explicit clarifies that the proposed framework should be understood not as an aggregation of separately beneficial technologies, but as a coherent response to a specific set of unresolved methodological constraints in contemporary bioinformatics workflows.

#### Pillar I without Pillars II and III: the passive representation problem

Foundation models for single-cell and multi-omic biology, such as scGPT [20], Nicheformer [21], and EpiAgent [22, 23], produce high-quality continuous representations of biological entities that support cross-tissue transfer, perturbation inference, and regulatory feature learning. However, when deployed in isolation these models remain passive epistemic artifacts: they transform inputs into embeddings or predictions, but they cannot autonomously determine which dataset to query next, which hypothesis to prioritize, or whether their outputs remain reliable when applied to data generated under previously unseen experimental conditions. The representational power of foundation models therefore does not, by itself, confer the capacity for multistage scientific reasoning, tool invocation, or iterative self-correction that defines agentic operation.

Furthermore, without a systematic validation layer, the robustness of these representations cannot be guaranteed. Empirical benchmarking studies have documented substantial performance degradation for biological foundation models under cross-platform transfer, preprocessing variability, and rare-population evaluation [20–24]. In the absence of Pillar III, such failure modes remain largely undetected within downstream workflows, including agentic workflows that rely on foundation model embeddings as their representational substrate. A foundation model integrated into an agentic pipeline without continuous validation can therefore propagate embedding instability as apparently confident biological hypotheses across multiple reasoning steps, amplifying rather than correcting the original artifact. The main failure modes arising from the removal of each pillar are summarized in Table 1, providing a concrete view of the operational risks in biomedical workflows.

**Table 1:** Failure mode analysis for each pillar when removed from the integrated architecture.

**Pillar removed**	**Functional capacity lost**	**Concrete failure mode in biomedical context**	**Supporting evidence/literature anchor**
Foundation models	No biologically grounded continuous representation of omic signals; agents must reason over text proxies (gene names, pathway labels, literature abstractions).	(i) Cross-platform transfer fails silently: without calibrated embeddings, label harmonization artifacts mimic generalization. (ii) Rare cell populations and regulatory states become invisible to text-level reasoning. (iii) Multi-omic tasks require shared latent geometry for cross-modal alignment, which cannot be achieved without representation learning.	Performance degradation under cross-platform transfer documented for scGPT [20], Nicheformer [21], EpiAgent [22, 23]; zero-shot limitations [22]; cross-site benchmarking studies [24, 25].
Multi-agent systems	No decomposition, orchestration, or tool-mediated execution; foundation model outputs remain static predictions disconnected from multistage discovery logic.	(i) A foundation model can embed a cell but cannot autonomously decide which dataset to analyze next, retrieve updated literature, or propagate outputs to downstream statistical tests. (ii) Conflicting signals across modalities (e.g. regulatory networks versus docking predictions) cannot be adjudicated without specialized agents. (iii) Without shared memory and provenance tracking, end-to-end workflows are not reproducible or auditable.	ReAct framework [17]; orchestrator-based MAS architectures [26–28]; MCP-governed tool ecosystems [29–31]; provenance requirements for computational reproducibility [25, 32].
Automated validation	No operational mechanism to detect silent error propagation, embedding instability, or statistically unreliable intermediate outputs across pipeline steps.	(i) Multi-step pipelines accumulate type-I errors without sequential testing checkpoints. (ii) Cross-platform embedding drift remains undetected without robustness benchmarks. (iii) Clinical or regulatory contexts require calibrated uncertainty estimates, rollback policies, and versioned audit trails. (iv) Conflict resolution between heterogeneous agent outputs requires executable validation rules rather than ad-hoc heuristics.	Biomedical reproducibility crisis [19]; benchmarking frameworks for foundation models [24–26]; sequential statistical testing requirements in iterative workflows [33, 34]; regulatory constraints for AI deployment in biomedical contexts [29, 35].

#### Pillar II without Pillars I and III: the representational mismatch and safety problem

Multi-agent architectures provide the orchestration, role specialization, tool-mediated execution, and provenance tracking necessary for end-to-end scientific workflows [17, 26–28]. However, agentic systems that lack biologically grounded foundation model representations face a fundamental mismatch when operating on high-dimensional omic data. General-purpose language model reasoning, even when augmented with sophisticated tool use, operates primarily at the level of textual abstractions: gene names, pathway labels, ontology identifiers, and literature descriptions. These proxies cannot capture the continuous representational geometry of single-cell expression matrices, epigenomic accessibility profiles, or spatially resolved transcriptomics. For tasks requiring cross-modal alignment, cross-species transfer, or the detection of subtle regulatory signals in rare cell populations, the absence of calibrated biological embeddings constitutes a structural barrier to reliable inference rather than a minor limitation.

Equally critically, multi-agent architectures without automated validation lack the operational safety mechanisms required for deployment in regulated biomedical contexts. Multistep agentic pipelines are inherently susceptible to error accumulation: a literature agent may retrieve an imprecisely matched study; an omics pipeline agent may apply a suboptimal preprocessing threshold; a drug–target agent may query an outdated database version. Without Pillar III, sequential statistical testing, executable precondition checks, and structured audit trails, these incremental errors propagate silently through the reasoning chain, producing outputs that may appear confident and internally consistent while resting on unreliable intermediate conclusions.

#### Pillars I and II without Pillar III: the reproducibility and regulatory gap

The combination of foundation model representations and multi-agent orchestration significantly extends the capabilities of bioinformatics AI systems. Nevertheless, even this two-pillar configuration remains insufficient for the most demanding applications, particularly those characterized by regulatory requirements, clinical translation goals, or high-stakes hypothesis generation from sparse data. The central limitation is operational: without a dedicated validation layer, there is no systematic mechanism to verify that the outputs of the combined system are statistically robust, reproducible across laboratories and preprocessing pipelines, or safe to act upon.

This concern is not merely theoretical. The biomedical reproducibility crisis [19] has demonstrated that multistage analytical pipelines can generate apparently compelling but non-replicable results even when each individual component is carefully designed. Agentic pipelines, by virtue of their greater complexity and the larger number of sequential decisions they embed, may be even more susceptible to such issues if systematic validation is absent.

Furthermore, in drug discovery and clinical contexts, the absence of structured validation constitutes a categorical barrier to deployment. Regulatory frameworks require traceable, versioned, and externally auditable decision processes [29, 35]. Without executable validation rules linked to audit trails, without sequential significance budgets that prevent type-I error inflation across iterative testing cycles, and without uncertainty-calibrated outputs that clearly distinguish between confident and uncertain inferences, even highly sophisticated foundation model–agent systems cannot satisfy the evidentiary standards required for clinical or regulatory use.

#### The three-pillar integration: necessity, scope, and what it achieves

The arguments above establish that each pairwise combination of pillars remains insufficient for a well-defined class of bioinformatics discovery tasks: those characterized by multistage, multimodal workflows operating on heterogeneous datasets under requirements of reproducibility, traceability, and regulatory safety. For this class of problems, the three-pillar integration is therefore not merely advantageous but constitutive of the workflow itself. Removing any single pillar produces a distinct structural failure: without Pillar I, the system cannot reliably represent complex biological signals; without Pillar II, it cannot orchestrate the multistage discovery process; and without Pillar III, it cannot guarantee the statistical reliability and regulatory acceptability of its outputs.

Importantly, not all bioinformatics applications require all three pillars simultaneously. Simpler workflows, such as protocol automation, literature synthesis, or monolithic toxicity classification, may be adequately supported by one or two components of the architecture. The defining use cases of the proposed paradigm, however, including rare-disease multi-omic drug repurposing, closed-loop personalized medicine, and autonomous multi-omic hypothesis generation, are precisely those for which the absence of any single pillar produces an unresolvable operational limitation. These applications also represent areas of particularly high scientific and clinical impact, providing the primary motivation for the integrated framework proposed in this review. Representative bioinformatics applications and the degree to which they require each pillar are mapped in Table 2, highlighting the scenarios where all three pillars are indispensable.

**Table 2:** Pillar necessity matrix for representative bioinformatics applications.

**Application/use case**	**Foundation models**	**Multi-agent systems**	**Automated validation**	**All 3 required?**	**Rationale**
Protocol automation/assay setup	○ Optional	✓ Required	~ Partial	No — 1–2 pillars core	Text-based RAG retrieval and procedural orchestration are sufficient; omic embeddings are not required. Basic output QC suffices.
Literature synthesis/prior-art analysis	○ Optional	✓ Required	~ Partial	No — 1–2 pillars core	Retrieval and summarization are primarily text-domain tasks. Foundation models may add semantic clustering but are not necessary.
*In vitro* toxicity triage	~ Partial	✓ Required	✓ Required	No — 1–2 pillars core	Molecular embeddings improve predictive models, but descriptors partially compensate. MAS orchestration and risk calibration remain essential.
Cross-platform single-cell annotation	✓ Required	~ Partial	✓ Required	No — 1–2 pillars core	Robust transfer across technologies requires calibrated embeddings; benchmarking and validation remain essential.
Rare-disease multi-omic drug repurposing	✓ Required	✓ Required	✓ Required	YES — jointly necessary	Sparse heterogeneous data require representation learning; MAS orchestration integrates literature, omics, and drug databases; validation prevents statistical artifacts on low-N datasets.
Closed-loop personalized medicine	✓ Required	✓ Required	✓ Required	YES — jointly necessary	Patient-specific multimodal integration requires FM embeddings; MAS coordination manages guidelines and data streams; AV ensures regulatory compliance and uncertainty calibration.
Autonomous multi-omics hypothesis generation	✓ Required	✓ Required	✓ Required	**YES — jointly necessary**	Cross-modal alignment, iterative hypothesis generation, and statistical error control require the full architecture.
Closed-loop automated synthesis	~ Partial	✓ Required	✓ Required	No — 1–2 pillars core	Laboratory orchestration and QC loops are essential; molecular embeddings improve route scoring but are not strictly required.

Finally, it is important to acknowledge that no currently published system has yet achieved the complete three-pillar integration within a prospectively validated biomedical context. The goal of this review is therefore not to report a solved engineering problem, but to articulate and map the architecture required to solve it. This approach is consistent with the methodological role of paradigm-defining reviews in computational biology and artificial intelligence: influential works have historically articulated conceptual frameworks before their full empirical realization. The framework described here, including the spinal muscular atrophy (SMA) workflow introduced in the following sections serves an analogous function, outlining the operational logic of the three-pillar integration, mapping existing tools and frameworks to each component, and identifying the engineering requirements that a complete implementation must satisfy. The historical analogy with prior paradigm-defining reviews is summarized in Table 3, situating the present three-pillar framework within the broader context of computational biology and AI methodology.

**Table 3:** Paradigm-defining review precedents in computational biology and AI.

**Reference**	**Paradigm proposed**	**State of empirical evidence at time of publication**	**Analogy to present review**
Bommasani et al. [7] (2021)	Foundation models as a general AI paradigm	GPT-3 existed, but large-scale biomedical deployments had not yet been realized; the paper articulated a conceptual framework rather than reporting domain implementations.	The present review similarly articulates the three-pillar paradigm before full biomedical realization.
Yao et al. [17] (2022)	ReAct (Reasoning + Acting) agentic framework	Demonstrated on web navigation and QA benchmarks; no biomedical or clinical validation existed at the time.	The SMA workflow introduced here serves an analogous role as an architectural demonstration rather than a prospective clinical deployment.
Lewis et al. [18] (2020)	RAG	Validated on NLP benchmarks; systematic biomedical applications emerged later.	RAG now constitutes a central component of validation and grounding mechanisms within the third pillar.
Jumper et al. [9] (2021)	Deep learning structural prediction paradigm	AlphaFold2 demonstrated unprecedented structural accuracy; downstream drug discovery and clinical translation pipelines were still emerging.	Analogously, the present review identifies an architectural paradigm whose full biomedical implementation represents the next research phase.

This end-to-end vision redefines bioinformatics as a discipline not only of biological data analysis, but of engineering the scientific process itself. If the first wave automated prediction and the second scaled representation, the third aims to automate, under constraints of rigor and transparency, substantial parts of the discovery cycle. The crucial challenge is no longer just technical, but methodological and regulatory: ensuring that increasingly autonomous agents remain interpretable, verifiable, and aligned with the principles of reproducible science.

## Universal representations and operational considerations in agentic systems

Foundation models are intended to serve as the “brain” of future agentic systems in bioinformatics, providing reusable and transferable embeddings capable of supporting complex multi-agent workflows. However, their practical utility depends not only on scale but also on the stability, generalization capacity, and reproducibility of the learned representations across heterogeneous biological datasets. Recent work suggests that architectural choices, such as generative versus contrastive pretraining objectives, introduce distinct trade-offs between representation flexibility, interpretability, and robustness to experimental variability [20–23, 36]. A recent systematic analysis of biological foundation models further emphasizes how model design, pretraining strategies, and downstream utilization shape their effectiveness across tasks [37]. Table 4 summarizes representative foundation models particularly relevant for agentic workflows. Although these systems share the goal of learning universal biological representations, they differ substantially in training objectives, input modalities, and assumptions about biological structure [26, 38]. Generative approaches such as scGPT aim to model gene expression distributions directly, enabling flexible downstream tasks including zero-shot annotation and perturbation inference. In contrast, models such as Nicheformer incorporate spatial context during pretraining, prioritizing the reconstruction of tissue microenvironments over purely transcriptional relationships. Phylogeny-aware architectures such as scPlantFormer explicitly encode evolutionary constraints to improve cross-species transfer, whereas epigenomic models such as EpiAgent emphasize regulatory feature learning across heterogeneous assay types.

**Table 4:** Representative foundation models for biological representation learning relevant to agentic AI workflows.

**Model**	**Type**	**Input modality**	**Training scale**	**Pretraining objective/approach**	**Key capabilities**	**Evaluation benchmarks/typical tasks**	**Known limitations/failure modes**
scGPT [20]	Generative Transformer	Single-cell RNA-seq	33 M+ cells	Autoregressive generative pretraining	Zero-shot annotation; cross-tissue transfer; gene–cell dependency capture	Cell-type annotation; cross-dataset transfer; perturbation response prediction; gene regulatory inference	Performance degradation under cross-platform transfer; sensitivity to preprocessing pipelines; reduced accuracy for rare cell populations
Nicheformer [21]	Spatial transformer	Single-cell + spatial transcriptomics	110 M cells (57 M dissociated +53 M spatial)	Contrastive/generative pretraining with spatial neighborhoods	Niche-aware inference; spatial context integration	Spatial domain identification; cell–cell interaction inference; tissue microenvironment reconstruction	Strong dependence on spatial resolution and neighborhood definition; limited generalization to dissociated datasets
scPlantFormer [36]	Phylogeny-constrained transformer	Plant single-cell RNA-seq	~1 M cells (Arabidopsis)	Phylogeny-constrained pretraining	Cross-species transfer; conserved regulatory logic alignment	Cross-species cell annotation; evolutionary conservation analysis	Limited training diversity outside model organisms; transfer performance decreases for distant species
EpiAgent [22, 23]	Epigenomic foundation model	ATAC-seq/epigenomic assays	Large-scale ATAC-seq data	Contrastive/supervised regulatory mapping	Regulatory logic modeling; cross-assay transfer; functional mapping	Chromatin accessibility prediction; regulatory element annotation; multi-omic integration	Sensitive to peak-calling strategies and assay-specific biases; cross-laboratory reproducibility challenges

The evaluation of basic biological models requires careful methodological design. The scale of the reported model, often expressed in millions of cells or sequences, is only meaningful if the training corpus reflects real biological and technical diversity. Otherwise, models risk learning artifacts specific to the dataset rather than generalizable biological principles. Empirical benchmarking studies have shown that performance improvements observed in standard evaluation settings can decrease substantially when models are tested across different laboratories, sequencing platforms, or preprocessing pipelines [20–23]. For example, cell type annotation tasks across different datasets often show significant performance degradation when training and evaluation datasets come from different experimental protocols. These findings suggest that model scale alone does not guarantee robust biological learning [26, 39].

Beyond technical variability, foundation models are also susceptible to systematic biases inherent in the underlying biological datasets. These may arise from uneven sampling of tissues, populations, or experimental conditions, as well as from preprocessing choices and annotation practices. Such biases can amplify errors in downstream analyses, propagate incorrect hypotheses through agentic pipelines, and compromise reproducibility across laboratories and platforms. Recognizing these risks is essential for designing evaluation protocols, stress-testing embeddings, and implementing safeguards before using representations in planning or decision-making modules. Complementary methodological criticalities and validation strategies are summarized in Table 5.

**Table 5:** Summary of key methodological challenges in foundation models for agentic bioinformatics systems.

**Challenge**	**Description**	**Typical evaluation strategies**	**Implications for agentic AI systems**
Zero-shot evaluation limitations	Apparent generalization may arise from dataset overlap, shared label ontologies, or harmonized preprocessing pipelines rather than true biological transfer [20, 22].	Strict dataset isolation; cross-laboratory validation; evaluation across independent cohorts.	Agents relying on fragile embeddings may propagate incorrect annotations or hypotheses across reasoning pipelines.
Dataset shift and cross-platform variability	Differences in sequencing technologies, preprocessing pipelines, and laboratory protocols introduce domain shifts that can degrade model performance [24, 40].	Cross-dataset benchmarks; platform-specific evaluation; domain generalization tests.	Planning modules may operate on embeddings that are unstable across institutions or datasets, reducing reliability of downstream analyses.
Reproducibility and preprocessing dependence	Small variations in normalization, gene filtering, or batch correction can alter learned representations and downstream predictions [24, 36].	Pipeline standardization; reproducibility studies across preprocessing workflows.	Lack of reproducibility can compromise auditability and traceability in multi-agent scientific workflows.
Interpretability and regulatory logic inference	Learned embeddings often capture statistical co-expression patterns rather than causal regulatory relationships [21, 36].	Perturbation-based validation; pathway enrichment tests; gene regulatory network benchmarks.	Limited mechanistic interpretability constrains the ability of agents to justify hypotheses or design targeted experiments.
Sparse and noisy omics data	Single-cell and epigenomic datasets are characterized by dropout events, compositional effects, and measurement noise [20, 22].	Robustness tests using simulated dropout or synthetic perturbations.	Agents may generate unstable hypotheses when operating on noisy embeddings without uncertainty calibration.

Zero-shot evaluation has become a popular metric for evaluating baseline models, but its interpretation requires caution. Apparent zero-shot performance can be influenced by subtle forms of data loss, label harmonization artifacts, or shared ontological mappings between datasets. When strict dataset separation is applied, recent analyses have reported notable drops in performance, particularly for rare cell populations or underrepresented biological conditions. Furthermore, differences in annotation standards across studies can artificially inflate reported accuracy when labels are retrospectively aligned. For these reasons, robust evaluation protocols increasingly emphasize cross-dataset transfer benchmarks, perturbation-based validation, and domain shift testing across laboratories and experimental platforms. Omics data pose unique challenges compared to language: single-cell RNA sequencing generates sparse count vectors with strong compositional effects; spatial transcriptomics mixes biological signals with capture efficiency that varies in space; epigenomic assays measure accessibility rather than expression, with peak definitions dependent on preprocessing choices. These properties motivate architectures that can handle data sparsity and noise, explicitly representing uncertainty and aligning multiple views of the same underlying biology. Universal representations are therefore best conceived as sets of interoperable embeddings and mappings that remain stable across technical variations rather than as single latent spaces. These properties also create characteristic failure modes for foundation models. Sparse expression matrices, technical dropout, and heterogeneous preprocessing pipelines can distort learned embeddings and lead to unstable downstream predictions, particularly when models are applied to datasets generated under previously unseen experimental conditions. Many models extend the vanilla transformer architecture to address these challenges, incorporating inductive biases such as sparse attention to reflect regulatory modularity, a priori graphs, or recurrent attention hybrids. The design trade-off balances expressiveness, stability, and sample efficiency, especially in regimes with limited labeled data and high measurement noise. Architectures optimized for broad transfer can underperform on niche but high-stakes tasks (e.g. minority-cell detection or rare regulatory states), whereas highly specialized models may provide stronger local accuracy at the cost of reduced portability across datasets. From an operational perspective, the robustness of these embeddings is critical because errors propagate through agentic pipelines. If foundational representations are unstable under small perturbations, downstream planning, tool invocation, and hypothesis generation modules may amplify these errors across multistep reasoning processes. Interpretability is an operational requirement in biomedicine: models must support concrete decisions, including target selection, perturbation design, biomarker prioritization, and hypothesis justification. This goes beyond generic feature attribution, requiring latent factors to be reproducibly linked to known pathways, normative schedules, or spatial niches. Evaluation should examine stability under resampling and batch changes, as well as concordance with external evidence such as known markers or responses to perturbations. Without operational interpretability, even highly predictive baseline models cannot function as the “brain” of a reliable scientific agent. Consequently, interpretability evaluation should not rely solely on qualitative inspection but should include quantitative stability tests, cross-dataset reproducibility analyses, and concordance with independent biological knowledge sources. These universal embeddings also enable closed-loop discovery: by preserving causal structure and quantifying uncertainty, agents can search for, propose, and test hypotheses more efficiently. In multi-agent workflows, models must support traceable and verifiable intermediate outputs. Practical readiness assessment involves integrating base models into tool-enhanced pipelines and measuring end-to-end robustness, evaluating consistency in the face of minor perturbations and the speed with which failures are detected and corrected. Overall, the emerging literature suggests that biological foundation models should be evaluated not only on predictive accuracy but also on reproducibility, cross-domain robustness, and stability under dataset perturbations. These criteria are particularly important when such models are embedded within agentic AI architectures, where errors in representation learning can propagate across planning, reasoning, and execution stages. Establishing standardized benchmarks and transparent evaluation protocols will therefore be essential for assessing whether current foundation models can reliably support autonomous or semi-autonomous scientific discovery workflows [26, 38, 41].

From an architectural perspective, biological foundation models should not be interpreted simply as another element of the toolset available to agentic systems. While many components in an agent pipeline implement task-specific operations (e.g. database queries, statistical analyses, or docking simulations), foundation models provide a more general representational substrate that supports reasoning, generation, and cross-modal inference across heterogeneous biological datasets. In this sense, they function less as individual tools and more as a shared cognitive layer upon which planning and action modules operate.

At the same time, the boundary between foundation models and agentic architectures may evolve. Recent biological foundation models already extend beyond representation learning, performing generative, reconstructive, and predictive tasks such as sequence generation, spatial reconstruction, or regulatory feature inference. As these systems increasingly incorporate tool use, iterative feedback, and structured reasoning loops, similar to the evolution observed in coding-oriented large language models, they may progressively acquire native agentic capabilities. Future architectures may therefore blur the distinction between “foundation model” and “agent,” with large multimodal biological models acting both as representation engines and as autonomous components within scientific discovery workflows.

## Frameworks for biomedical AI agents

**Figure 1:**
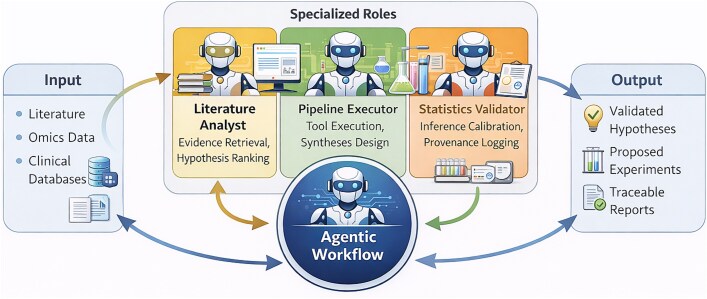
Agent-based workflow in drug discovery. A scientific goal (e.g. identifying a drug for a rare disease) is decomposed by an orchestrator agent into subtasks assigned to specialized agents. The literature analyst retrieves relevant publications via RAG; the pipeline executor accesses and analyzes public omic datasets; the statistical validator assesses result robustness through hypothesis testing and uncertainty quantification. A shared memory layer tracks all intermediate outputs, i.e. queries, parameters, results, enabling iterative refinement through reflection loops until verifiable, evidence-supported hypotheses are generated. This architecture operationalizes PARM framework in a distributed multi-agent setting.

Recent advances in biomedical artificial intelligence increasingly highlight the importance of systems capable of coordinating reasoning and tool use in complex, multistage workflows [17, 26, 38, 41]. Several recent studies have proposed agentic architectures that integrate reasoning, tool execution, iterative self-evaluation, and external memory to address complex biomedical tasks. To provide a systematic overview, we classify representative agent frameworks according to orchestration strategy, tool-governance design, and evidence-traceability mechanisms, as summarized in Table 3. This taxonomy complements our Planning–Action–Reflection–Memory (PARM) framework [17, 29, 39], situating our multi-agent workflows within the broader landscape of agentic AI while clarifying trade-offs in modularity, interoperability, and translational readiness. Importantly, PARM components should be interpreted as a conceptual decomposition of agentic functionality rather than as fixed software modules tied to a single architectural level. In practice, these components can be implemented either within a single agent or distributed across multiple specialized agents in a coordinated multi-agent system. In the latter case, different agents may predominantly implement specific functions (e.g. a literature agent performing action-oriented retrieval, a validation agent implementing reflection mechanisms, or an orchestrator agent responsible for planning and task decomposition). Figure 1 illustrates this distributed interpretation, where specialized agents collectively realize the PARM functions across the workflow while sharing a common memory and coordination layer.

The four components allow for a structured comparison of heterogeneous agents, whose maturity varies substantially depending on study design, validation setting, and external reproducibility [26, 38, 41]: (i) planning encompasses cognitive processing, reasoning, and decision-making, such as synthesizing clinical notes, supporting differential diagnosis, or reasoning about clinical trial eligibility. (ii) Action implements decisions through controlled interfaces with external systems [29, 35], including electronic health record (EHR) application programming interfaces (APIs), laboratory instrumentation, or robotic platforms. (iii) Reflection monitors performance and uncertainty, enabling iterative error checking, conflict resolution, and replanning. Applications include medical image analysis, real-time patient monitoring, and consistency checks between results and hypotheses. (iv) Memory preserves context and tracks provenance via short-term working states and long-term retrieval mechanisms, such as RAG on institutional guidelines, literature, and knowledge bases [18, 32].

**Figure 2:**
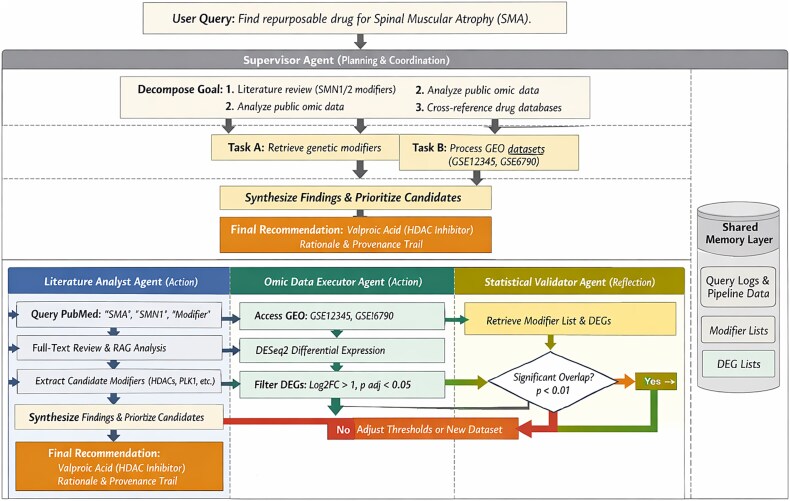
Multi-agent workflow for rare-disease drug repurposing. A supervisory planning/orchestrator agent decomposes the clinical objective (identifying a candidate drug for SMA) into a structured task graph. The literature analysis agent retrieves publications (e.g. via PubMed Entrez Programming Utilities, E-utilities), processes them through RAG pipelines, and stores structured outputs in a shared memory layer, while reflection monitors coverage and conflicts. The omics pipeline agent retrieves transcriptomic datasets from the GEO or ArrayExpress, performs preprocessing, differential expression, and pathway enrichment, with reflection evaluating stability across preprocessing variants. The validation/reflection agent integrates outputs, performs statistical consistency checks, uncertainty-aware aggregation, and sequential verification to reconcile conflicts across graph-based, molecular, or experimental predictions. All intermediate results are persisted with provenance, enabling reproducible, evidence-backed candidate prioritization (e.g. valproic acid) via cross-referencing with drug-target databases (e.g., DrugBank).

Beyond the functional decomposition into planning, action, reflection, and memory, the effectiveness of biomedical multi-agent systems depends critically on the mechanisms used to coordinate cooperation among agents. These mechanisms encompass organizational structure, communication protocols, and strategies for reaching consensus and resolving conflicts when agents produce divergent outputs. Multi-agent systems in biomedicine typically adopt hierarchical or orchestrator-based organizations, where a supervisory agent decomposes high-level objectives into subtasks and assigns them to domain-specialized agents such as literature analysts, omics pipeline executors, or statistical validators. This decomposition can be static, following predefined workflow templates, or dynamic, with the orchestrator adapting the task graph based on intermediate results. Figure 1 illustrates this structure, showing how the orchestrator coordinates specialized agents while maintaining a shared memory layer that persists throughout the workflow. Communication between agents is implemented through two complementary patterns. The first is structured message passing, where agents exchange explicit task requests, results, and control signals using defined protocols such as JSON schemas for task specifications, RESTful API calls, or specialized agent communication languages. The second is shared memory communication, where agents post intermediate outputs, provenance metadata, and status information to a common blackboard accessible to all agents. This shared memory approach, illustrated in Figs 1 and 2, offers particular advantages for traceability, as every read and write operation can be logged and versioned. Hybrid architectures combining both patterns are also observed, with shared memory used for persistent data and message passing for time-sensitive coordination. Because agents may operate on heterogeneous representations, molecular graphs, omics embeddings, literature-derived knowledge, structural models, or clinical data, conflicts between predictions are expected and must be managed explicitly. Multi-agent systems typically implement three primary strategies for resolving such conflicts, which are elaborated in the SMA workflow below. Confidence-weighted aggregation allows each agent’s output to be accompanied by a calibrated confidence score derived from statistical support, dataset coverage, or model uncertainty estimates, enabling the supervisory validator to integrate these scores into consensus rankings that down-weight hypotheses supported by weaker evidence. Cross-validation across independent evidence streams triggers additional analyses using alternative methods or datasets when conflicts arise, for instance, requesting docking simulations or literature-based mechanism checks when graph-based and molecular predictions disagree. Sequential verification subjects candidates that survive initial filtering to progressively stricter validation steps, with failures leading to deprioritization or exclusion. These mechanisms ensure that final recommendations reflect robust consensus rather than isolated predictions, with all resolution steps logged in the shared memory layer for full auditability. These coordination protocols map directly onto the PARM framework introduced earlier: planning encompasses task decomposition and assignment; action implements communication through tool interfaces and message passing; reflection houses consensus and conflict resolution algorithms; and memory maintains the shared provenance store that enables coordination, traceability, and *post-hoc* verification.

To clarify the strength of evidence underlying current agentic systems, we distinguish three general levels of maturity in the literature: (i) conceptual or architectural proposals, often reported in preprints or conference settings; (ii) controlled evaluations of prototypes, typically conducted in simulated environments, retrospective datasets, or sandbox tool suites; and (iii) externally validated or implementation-oriented studies with prospective or multisite evaluation. Most current biomedical studies fall within the first two levels, and only a limited subset provides robust external validation or analysis of generalizability across institutions [26, 38, 41].

Within this framework, planning operates as a strategic layer that breaks down complex objectives into sub-tasks that can be verified through heterogeneous biomedical data sources and analytical steps. The operational layer implements these plans through controlled interfaces, such as EHRs, laboratory information systems, or experimental automation platforms, where security constraints, authentication protocols, and rollback mechanisms are fundamental design requirements [29, 35]. Reflection provides continuous iterative error control, uncertainty monitoring, and dynamic replanning capabilities, while memory ensures context persistence and complete provenance tracking through a combination of short-term working states and long-term retrieval mechanisms [17, 18, 32].

Table 6 provides a complementary perspective, showing how different agent framework families implement these planning, action, reflection, and memory components in practice, highlighting orchestration strategies, tool governance, and inter-agent coordination. This taxonomy clarifies why different framework families are preferable for different discovery objectives and risk profiles, while making explicit the trade-offs between modularity, interoperability, and translational readiness.

**Table 6:** Comparative taxonomy of agent framework families in biomedical AI.

**Framework family**	**Representative frameworks**	**Typical orchestration pattern**	**Typical limitations/translational constraints**	**Primary strengths**
ReAct-style tool-using agents	ReAct [17]	Single-agent reasoning–action loop with iterative tool calls	Limited role specialization; brittle long-horizon planning; weak native support for multi-agent disagreement handling	Simple control flow; low setup overhead; strong baseline for retrieval and sequential decision tasks
Conversation-centric agent teams	AutoGen, CAMEL [27, 42]	Supervisor-worker or peer dialogue among specialist agents	Coordination overhead; prompt-protocol instability; increased risk of error propagation across agent messages	Modular specialization; scalable task decomposition; flexible inter-agent critique
Predefined role graph agents	Role-engineered agent teams; MetaGPT [28]	Predefined role graph with standard operating procedures for each agent	Higher engineering complexity; portability depends on task-template fit; limited validation in biomedical prospective settings	Better process standardization; clearer responsibility boundaries; improved reproducibility versus *ad hoc* prompting
Middleware-governed tool ecosystems	ChemCrow, Coscientist; MedCP-oriented architectures [29–31]	Agent layer decoupled from tools via standardized protocol adapters; multistep planning with chemistry-specific tools and experimental feedback loops	Immature ecosystem; additional infrastructure burden; security and authorization policies remain implementation-dependent; end-to-end hypothesis-to-experiment safety/regulatory barriers	Strong interoperability; auditable tool invocation; easier integration with heterogeneous systems; direct linkage between reasoning and lab execution

To illustrate how the PARM paradigm can be instantiated in a realistic computational context, Fig. 2 and Table 7 present a detailed multi-agent workflow for drug repositioning in SMA, a rare genetic disease characterized by progressive motor neuron degeneration caused by alterations in the SMN1/SMN2 pathway. The example makes explicit the operational logic of a coordinated agent system, including task decomposition, data exchange between agents, representative API interactions with biomedical data sources, and reflection mechanisms that regulate iteration and quality control.

**Table 7:** Operational mapping of agentic components to a reproducible drug-repurposing pipeline.

**Stage**	**Main component**	**Data flow (input → output)**	**Representative API/tool calls**	**Reflection and control process**
Goal decomposition	Planning (orchestrator)	Clinical objective + constraints → executable task graph	POST/planner/decompose; guideline retrieval via RAG; ontology normalization services	Verify constraint completeness, detect conflicting objectives, and assign initial uncertainty estimates before execution
Evidence aggregation	Action (literature agent) + Memory	Task graph nodes → ranked evidence packets with provenance links	GET/pubmed/search; GET/clinical trials/query; vector DB retrieval and reranking	Trigger re-query if coverage is low; remove duplicate/contradictory evidence; update memory with source-level confidence
Omics processing	Action (pipeline agent)	Public omics datasets → disease signatures and candidate mechanism scores	GET/geo/query; preprocessing + differential analysis workflow; pathway enrichment tools	Run QC and stability checks across preprocessing variants; if instability is detected, rollback and reprocess
Candidate prioritization	Planning + reflection	Integrated evidence + signatures → ranked repurposing candidates and rationale traces	Drug-target graph queries; interaction filters (GET/drugbank/interactions); ranking service API	Compare independent agent rankings; flag disagreement; down-weight candidates with weak mechanistic coherence
Validation loop	Reflection (validator agent) + memory	Top candidates → accepted candidates + uncertainty report + audit log	POST/validator/check; bootstrap/sensitivity testing; versioned run logging	Enforce stopping rules (minimum evidence support, calibration bounds); iterate back to prior stages or finalize outputs

In this scenario, a supervisory agent (or orchestrator) receives the high-level clinical objective, i.e. to identify drug candidates for repositioning for SMA, and breaks it down into a structured graph composed of sequential and parallel subtasks. In the planning phase, the system translates the initial objective into a series of concrete analytical operations, such as retrieving known genetic modifiers and signaling pathways associated with SMN1/SMN2 genes, the analysis of transcriptomic datasets derived from SMA patient samples or disease models, and the cross-checking of the resulting disease signatures with drug databases to identify compounds that target the relevant pathways.

Once the activity graph is defined, specialized agents perform the corresponding analytical steps through controlled interactions with external data sources and computational tools. A literature analysis agent performs structured queries on biomedical archives such as PubMed through the Entrez E-utilities interface and retrieves relevant publications and clinical reports. The retrieved documents are processed through retrieval-enhanced generation pipelines operating on full-text corpora, enabling the extraction and classification of disease modifiers, pathway associations, and potential therapeutic mechanisms reported in the literature. In parallel, an omics pipeline agent accesses public transcriptomic repositories such as Gene Expression Omnibus (GEO) or ArrayExpress to retrieve SMA-related datasets and perform standardized preprocessing and differential expression workflows. For example, transcriptomic profiles derived from patient motor neurons can be processed to identify differentially expressed genes and enriched pathways that characterize the disease state.

The intermediate results generated by these agents are stored in a shared memory layer, which serves as a persistent coordination substrate for the entire system. This layer maintains versioned records of retrieved articles, processed gene expression matrices, extracted biological entities, pathway enrichment scores, and drug-target associations obtained from pharmacological databases such as DrugBank. By preserving complete provenance information, including query parameters, data sets used, and analytical thresholds, the memory component ensures traceability and reproducibility throughout the workflow, allowing the supervisory agent to reconstruct the chain of reasoning leading to a given therapeutic hypothesis.

The reflection component continuously evaluates the consistency and statistical robustness of intermediate results. In multimodal discovery settings, reflection also plays a central role in resolving conflicts between heterogeneous inference sources. In practice, drug discovery pipelines frequently combine agents operating on different representational spaces, such as graph-based network models capturing drug–target or pathway interactions and molecular-level models based on sequence, structural, or physicochemical features. Because these approaches rely on distinct inductive biases, they may occasionally generate contradictory hypotheses regarding candidate compounds or mechanisms of action. To address this situation, reflection modules typically implement algorithmic conflict-resolution strategies. One common approach relies on confidence-weighted aggregation, in which each agent produces a calibrated confidence score derived from statistical support, dataset coverage, or model uncertainty estimates. The supervisory validator then integrates these scores to generate a consensus ranking, down-weighting hypotheses supported by weaker or less reproducible evidence streams. A complementary strategy is sequential verification, where conflicting hypotheses trigger additional targeted analyses before final prioritization. For example, if a graph-based agent identifies a candidate drug through network proximity to disease genes while a molecular modeling agent predicts poor binding affinity or structural incompatibility, the reflection module can request further validation steps. These may include additional docking simulations, independent omics validation, literature-based mechanism checks, or cross-dataset reproducibility tests. Hypotheses that consistently fail sequential verification are deprioritized or discarded. More generally, the reflection layer functions as a consensus-building and arbitration mechanism across heterogeneous analytical agents. By combining uncertainty-aware weighting, iterative verification, and provenance tracking, multi-agent systems can reconcile partially conflicting signals derived from network biology, molecular modeling, and experimental datasets while maintaining transparency regarding how final therapeutic hypotheses are produced.

A dedicated validation agent integrates lists of modifiers derived from the literature with differentially expressed genes identified by the omics pipeline and performs statistical analyses such as overrepresentation tests to identify significant overlaps between the two streams of evidence. The resulting gene sets are then cross-referenced with drug-target interaction databases to identify compounds whose mechanisms of action intersect with the detected disease pathways. When inconsistencies arise, such as when pathways derived from the literature do not significantly overlap with omics-derived disease signatures, the reflection module can trigger iterative correction steps. These may include adjusting statistical thresholds, incorporating additional datasets, or issuing new bibliographic queries to increase evidence coverage.

Through this iterative cycle of reasoning, heterogeneous evidence streams (biological mechanisms derived from the literature, transcriptomic signatures of diseases, and drug interaction networks) are progressively integrated to produce a ranked list of reusable candidate compounds. The final prioritization phase combines measures of statistical significance, mechanistic plausibility, and consistency across datasets to generate structured recommendations accompanied by uncertainty estimates and provenance records. For example, a compound such as valproic acid may emerge as a candidate due to its known HDAC inhibitory activity and the observed overexpression of related pathways in multiple transcriptomic datasets.

The design of this workflow highlights several engineering considerations that are fundamental to robust agent-based biomedical systems. First, the action layer must coordinate heterogeneous APIs and data formats, such as XML outputs from PubMed queries or MINiML metadata from GEO repositories, which require standardized parsing routines and error handling mechanisms to maintain reliability in interactions between tools. Second, reflection mechanisms operate continuously rather than as a final validation step, using both quantitative indicators (e.g. statistical significance thresholds) and qualitative signals (e.g. conflicting evidence in the literature) to trigger further analytical iterations. Finally, conscious provenance memory functions as a first-class component of the architecture, recording every query, parameter choice, and intermediate output so that the entire analytical trajectory can be reproduced and verified. This provenance structure not only supports reproducibility, but also enables diagnostic analysis of intermediate steps, allowing both agents and human researchers to verify how the final therapeutic hypotheses were generated.

## Use cases of agentic AI in bioinformatics

The paradigm of foundation models integrated with agentic systems extends across a much broader range of bioinformatics domains. In evolutionary genomics, agentic pipelines can coordinate large-scale comparative analyses, automatically integrating genome alignments, phylogenetic inference, and functional annotation across thousands of species, while reflecting on inconsistencies between sequence-based and structure-based evolutionary signals [4]. In structural biology, agents may orchestrate structure prediction, molecular docking, and binding affinity estimation, iteratively validating results against experimental databases and triggering re-runs when confidence thresholds are not met [8, 9]. Multi-omics integration represents another natural application area, where specialized agents analyze transcriptomic, epigenomic, proteomic, and spatial datasets, reconciling heterogeneous signals through shared representation spaces, a task particularly suited to foundation models capable of cross-modal alignment [12, 43, 44]. In spatial omics, agentic workflows could integrate imaging data, spatially resolved transcriptomics, and histological annotations to reconstruct tissue microenvironments, with reflection modules monitoring consistency across spatial resolutions and batch effects [16, 21]. More broadly, any bioinformatics workflow characterized by multistage data processing, heterogeneous evidence sources, and iterative hypothesis testing, including pathway analysis pipelines, variant interpretation workflows, functional enrichment analyses, and systems biology model construction, can benefit from agent-based orchestration supported by foundation models [2, 10]. The common thread is the integration of diverse tools and data types while maintaining auditability, precisely the strength of the multi-agent paradigm.

Among these domains, drug discovery is rapidly moving from conceptual experimentation to structured implementation in early- and mid-stage research and development workflows. Recent analyses of AI-based discovery pipelines highlight measurable reductions in comprehension time, improved knowledge synthesis, and increased reproducibility when reasoning systems are paired with tool-mediated execution and provenance tracking. Rather than functioning as isolated predictive models, agentic architectures coordinate literature retrieval, hypothesis generation, safety assessment, and iterative design-test cycles within verifiable multistage workflows.

The representative applications summarized in Table 5 highlight that the most immediate impact of agentic AI lies in compressing the information bottleneck that characterizes the early phase of drug discovery. Target identification and state-of-the-art analysis traditionally require manual synthesis of heterogeneous evidence spanning pathway biology, medicinal chemistry, and pharmacology. Multi-agent systems address this bottleneck by combining retrieval-enhanced generation with role specialization, in which sub-agents focus on distinct domains (e.g. mapping mechanisms of action, profiling adverse events, structural chemistry) under the supervision of an orchestrator [33, 45, 46]. By explicitly tracing the provenance of evidence and ranking candidate hypotheses, such systems can reduce the time required for structured review from weeks to hours, while preserving verifiability and reproducibility.

In preclinical development, agentic systems increasingly integrate reasoning cycles with controlled instrument execution. Toxicity and safety triage workflows often follow a ReAct-type paradigm, in which iterative reasoning is interspersed with database queries, simulation tools, and structured checkpoints [33, 47]. Agents can propose *in vitro* tests, retrieve historical toxicity data, update probabilistic risk profiles, and flag uncertain cases for expert evaluation. Reported implementations suggest a substantial increase in productivity in screening and triage cycles when reasoning and execution levels are closely correlated [45, 47]. Importantly, these architectures maintain traceable decision paths, addressing concerns about reproducibility and governance that have historically limited automated preclinical pipelines.

Protocol automation represents another high-value application area. Routine laboratory procedures, such as qPCR setup, compound dilution planning, and assay calibration, can be reformulated as retrieval-based procedural synthesis tasks. In this model, the agent retrieves validated experimental templates, verifies constraints (instrument model, reagent availability, temperature cycles, volume thresholds), and generates executable checklists suitable for supervised validation [33, 46]. This approach shifts the work from manual model modification to structured supervision, reducing setup times by orders of magnitude in reported cases while improving standardization across laboratories [45].

Workflows related to drug repositioning highlight a complementary strength of agent-based AI: the systematic aggregation of fragmentary evidence. Rare diseases and niche indications often suffer from a scarcity of data distributed across clinical cases, molecular signatures, and small cohort studies. Supervisor-based multi-agent architectures can independently search, classify, and reconcile candidate mechanisms and compounds while maintaining explicit chains of provenance [27, 34, 48]. Although such systems do not replace clinical validation, they can prioritize mechanistically plausible candidates for downstream experimental testing, improving allocation efficiency in resource-constrained research environments.

The integration of *in silico* reasoning with *in vitro* execution further expands the scope of agentic systems. Emerging “AI organic chemist” platforms combine synthesis planning algorithms with laboratory automation interfaces, enabling partial or closed-loop experimentation [34, 47]. In these systems, planning modules generate synthetic pathways, action layers translate them into instrument-level instructions, and analytical test feedback informs subsequent iterations. Even when full autonomy is limited by regulatory or safety requirements, partial automation of orchestration, job scheduling, and quality control verification improves reproducibility by ensuring that identical inputs produce reproducible execution sequences.

Personalized medicine introduces an additional layer of complexity by incorporating patient-specific multimodal data streams. However, integrating heterogeneous biomedical modalities can lead to partially conflicting inferences when models operating on different representations, such as graph-based drug–target networks, transcriptomic signatures, and molecular sequence or structural models, propose divergent therapeutic hypotheses. In practical multi-agent implementations, such discrepancies are handled within the reflection layer through consensus-building mechanisms. These typically include confidence-weighted aggregation of agent outputs, where predictions are weighted according to statistical support or model uncertainty, as well as sequential verification strategies in which conflicting candidates trigger additional validation steps (e.g. cross-dataset replication, docking simulations, or literature-based mechanism checks). Through this iterative arbitration process, multi-agent systems can reconcile heterogeneous evidence streams while maintaining transparent provenance of the decision path leading to the final therapeutic recommendation.

Within this context, generative modeling frameworks provide a complementary capability by exploring large hypothesis spaces while respecting the constraints imposed by heterogeneous biomedical evidence. Generative modeling frameworks, which often integrate sequence and graph transformers with latent variable representations, are increasingly used to propose individualized therapeutic hypotheses or optimized drug combinations in the presence of explicit clinical constraints [45, 46]. In this context, agentic architectures primarily provide coordination: aligning multi-omic preprocessing, evidence retrieval from guidelines and literature, model-based hypothesis generation, and validation pipelines into verifiable governance frameworks. Rather than replacing clinicians, these systems function as structured cognitive amplifiers, synthesizing heterogeneous evidence while preserving transparency and traceability.

The use cases in Table 8 illustrate a converging architectural pattern: orchestrator-based multi-agent coordination, retrieval-based reasoning, structured tool integration, and verifiable memory layers. The central engineering challenge is not only predictive accuracy, but also reliable orchestration across heterogeneous tools, data modalities, and regulatory constraints. As recent implementations indicate, the value of agentic AI in drug discovery and personalized medicine emerges when reasoning, action, and evidence monitoring operate as an integrated system capable of accelerating insight without compromising reproducibility or governance [27, 33, 34, 45–48].

**Table 8:** Representative use cases of agentic AI in drug discovery and personalized medicine.

**Application**	**Framework pattern**	**Operational role of agentic components**	**Reported/expected quantitative impact (with refs.)**
Molecular literature and prior-art analysis	Orchestrator + specialist sub-agents + RAG	Planning: hypothesis decomposition and query design; Action: structured literature/database retrieval; Reflection: cross-source consistency checking; Memory: provenance tracking and citation linking	Weeks → hours for structured evidence synthesis; improved auditability and traceability in multi-agent biomedical reasoning workflows
*In vitro* toxicity and safety triage	ReAct-style iterative reasoning + tool calls + human-in-the-loop checkpoints	Planning: propose assays/tests; Action: query toxicology databases and predictive models; Reflection: update risk profiles and flag uncertainty; Memory: cumulative safety state	~10× productivity in iterative triage loops; structured escalation with human oversight in healthcare agent architectures
qPCR/assay protocol automation	RAG + constraint checking + executable checklist generation	Planning: protocol synthesis under reagent/instrument constraints; Action: lab-system interfacing; Reflection: validation of volumes, cycles, thermocycler compatibility; Memory: version-controlled procedural archive	Orders-of-magnitude reduction in setup time and improved procedural reproducibility in tool-augmented agent frameworks
Rare-disease drug repurposing	Supervisor architecture with multiple domain-specialist sub-agents + evidence aggregation	Planning: candidate prioritization under sparse data; Action: heterogeneous evidence retrieval (case reports, omics, small studies); Reflection: mechanistic reconciliation and conflict resolution across heterogeneous evidence streams (e.g. confidence-weighted aggregation of network- and omics-derived signals; sequential verification through additional datasets or literature evidence); Memory: explicit provenance chains	Improved ranking of mechanistically plausible candidates through coordinated multi-agent evidence aggregation
Automated molecular synthesis (“AI organic chemist”)	Planning modules + retrosynthesis generators + lab automation interfaces	Planning: route optimization and feasibility scoring; Action: instrument-level execution and job orchestration; Reflection: analytical feedback integration; Memory: experiment logs and replayable workflows	Closed-loop synthesis and assay execution with enhanced reproducibility and auditable experiment histories
Personalized multi-omics therapeutic hypothesis generation	Generative modeling + multi-agent coordination + governance layer	Planning: patient-specific constraint modeling; Action: guideline and literature retrieval; Reflection: multimodal validation and arbitration of heterogeneous model outputs (e.g. confidence weighting of graph-based and molecular predictions, sequential verification across independent datasets); Memory: longitudinal patient representation	Coordinated, auditable generation of individualized therapeutic hypotheses in biomedical agent systems

### Automated validation system

The third pillar of the proposed paradigm concerns automated validation systems, i.e. explicit mechanisms that verify whether intermediate and final results remain statistically and operationally reliable during multi-agent execution. In practice, validation is not a single final check, but a continuous control process tightly coupled with planning, tool invocation, and memory updates. For biomedical workflows, this level should be implemented as executable rules linked to audit trails, so that every decision to accept, reject, re-request, or reschedule is traceable and reproducible [26, 33, 34].

From an operational perspective, automated validation can be structured on three levels. The first level is input and protocol validation, which includes schema checks, ontology consistency, threshold verification, and prerequisite validation for each analytical tool (e.g. minimum sample size, compatible normalization, required covariates). The second level is evidence and inference validation, where retrieved claims are cross-checked against citable sources, contradiction rates are monitored across agents, and reliability updates are constrained by predefined rules. The third level is output and implementation validation, where candidate hypotheses are stress-tested under perturbation, compared across independent test channels, and released only if they meet predefined safety and calibration criteria. This layered structure reduces silent failure modes and clarifies which module is responsible in the event of a pipeline failure.

Sequential statistical testing is a fundamental component of this pillar because agentic pipelines are inherently iterative. Rather than performing a single end-of-pipeline check, systems evaluate evidence at planned checkpoints (after retrieval, after omic integration, after candidate classification) using explicit continuation and termination policies. In practical terms, continuation should require consistent evidence growth across iterations, while rollback or escalation should be triggered by instability in resampling, calibration drift, degraded out-of-distribution behavior, or disagreement between orthogonal models (e.g. graph-based network predictors versus molecular-level embeddings).

In these contexts, hybrid frameworks that combine graph-based reasoning with molecular or structural feature modeling require explicit contradiction-management policies to remain operationally reliable. When specialist agents disagree, confidence-weighted aggregation should be coupled with sequential verification on orthogonal evidence, such as pathway coherence checks, independent assay signals, or external validation cohorts. Persistent conflicts should trigger an “uncertain” status with escalation to expert adjudication rather than forcing consensus. This ensures both operational safety and auditability in translational decision pipelines, maintaining traceable provenance of the reasoning process.

To avoid inflated false positives in repeated tests, validation policies should predefine significance budgets, correction strategies, and maximum iteration limits. Benchmarking must align with this sequential logic. In addition to task-level accuracy, evaluation should report robustness to dataset shift, reproducibility across preprocessing pipelines, uncertainty calibration, and end-to-end verifiability of execution traces [24–26]. A minimally informative benchmark suite should therefore include: (i) cross-site or cross-cohort transfer; (ii) perturbation and ablation testing; (iii) variance and sensitivity analysis of repeated executions; and (iv) process metrics such as rollback frequency, unresolved conflict rate, and percentage of decisions subject to human review. In this framework, automated validation serves as a core operational governance mechanism that determines when an agent can proceed autonomously and when supervision is required.

## Discussion

Despite rapid progress, deploying agentic AI for end-to-end biomedical discovery and clinical translation remains constrained by a set of intertwined technical, organizational, and regulatory challenges. Unlike conventional predictive models, agentic systems do not merely generate outputs; they plan, retrieve evidence, invoke tools, and execute actions. This expanded operational scope amplifies both their potential impact and their risk surface.

A first critical challenge concerns reliability and hallucination propagation. In agentic systems, hallucinations are not limited to textual inaccuracies; they can influence planning, distort evidence aggregation, and ultimately trigger incorrect tool-mediated actions. RAG reduces but does not eliminate this risk, as failures may arise from flawed retrieval ranking, misinterpretation of correctly retrieved sources, or brittle orchestration between planning and execution layers. Moreover, multi-agent coordination introduces additional failure modes, including inconsistent state representations or unverified intermediate conclusions. Addressing these risks requires multilayer safeguards: citation validation against primary sources, executable precondition checks (e.g. verifying statistical assumptions before initiating analyses), structured logging of tool calls, and versioned audit trails that enable *post-hoc* traceability and root-cause analysis. In high-stakes biomedical contexts, robustness must be engineered across the entire reasoning–action pipeline rather than evaluated solely at the output layer.

These reliability concerns point to a deeper architectural issue: many of the observed failure modes arise not simply from individual components but from the absence of a tightly integrated system capable of coordinating representation, reasoning, and validation. The necessity argument developed in the Introduction established the analytical basis for the integrated paradigm; here, we consider what such integration achieves in practice beyond the capabilities of any two of its components. This question can be illustrated through the SMA drug repurposing workflow presented earlier in the paper, which serves as an existence proof of the integration logic in a realistic biomedical context.

For the class of tasks that define the proposed paradigm, i.e. sparse-data, multimodal, multistage, and regulated workflows, the value of integration is not merely additive but multiplicative. This is because the failure modes associated with different architectural components compound in ways that cannot be resolved by the remaining pillar alone.

Consider the SMA repurposing workflow. A system combining foundation model embeddings with multi-agent orchestration could in principle generate a ranked list of candidate compounds based on transcriptomic signatures and literature evidence. Yet without automated validation there is no operational mechanism to determine whether the transcriptomic signatures supporting the ranking remain stable across the heterogeneous and sparse SMA datasets available in public repositories, whether the statistical tests used to generate significance scores appropriately control for the multiple comparisons implicit in an iterative analytical pipeline, or whether drug–target associations retrieved from pharmacological databases are supported by independent evidence streams rather than relying on a single poorly replicated study. In the absence of such validation mechanisms, the system’s confident output, a ranked list of candidates with apparent mechanistic support, may rest on a compounding chain of implicit assumptions that no individual component can surface or correct.

A different but equally fundamental limitation arises in a system that combines multi-agent orchestration with automated validation but lacks biologically grounded foundation model representations. SMA-related transcriptomic datasets are sparse, generated across heterogeneous platforms, and characterized by the high technical variability typical of rare-disease research. Without calibrated biological embeddings, agent-level reasoning over gene names, pathway labels, and literature abstractions cannot reliably distinguish genuine disease signatures from platform-specific artifacts, a failure mode that has been documented in cross-platform benchmarking studies of biological foundation models. In such a scenario, the validation layer may detect statistical instability in downstream outputs, but it cannot provide the missing representational capacity required to prevent that instability in the first place.

The integration of all three pillars addresses these limitations simultaneously. Foundation model embeddings provide a biologically calibrated representational substrate capable of stabilizing cross-platform transfer. Multi-agent orchestration decomposes the discovery task, coordinates heterogeneous computational tools, and maintains a shared provenance layer that records the sequence of analytical steps. Automated validation continuously monitors embedding stability, applies sequential statistical controls, and escalates uncertain candidates to expert review rather than forcing spurious consensus. The resulting system exhibits a qualitatively different operational capacity: a closed reasoning–action–validation loop in which each component constrains and amplifies the others, and in which errors that would propagate silently in a two-pillar architecture can instead be surfaced, localized, and corrected before influencing downstream conclusions.

Beyond this compounding-failure argument, the integrated architecture also produces positive synergies between its components. When foundation model representations are combined with multi-agent orchestration, different specialist agents can invoke modality-specific models and share their outputs through a common memory layer with normalized provenance metadata. The orchestrator can then align these heterogeneous representations without requiring a single monolithic model to handle all modalities simultaneously.

A further synergy emerges from the integration of automated validation with multi-agent orchestration. Validation rules can be embedded directly into the execution checkpoints of the agent workflow, ensuring that each analytical step is preceded by explicit precondition checks and followed by postcondition checks evaluating statistical robustness and reproducibility. In this configuration, validation becomes an intrinsic component of the reasoning loop rather than a retrospective audit.

Finally, the integration of foundation models with automated validation enables calibrated uncertainty propagation, which is particularly important in rare-disease and low-sample-size contexts. Embedding spaces produced by foundation models can be evaluated for stability under resampling or perturbation, providing a basis for estimating uncertainty that propagates through the entire analytical pipeline.

It is important, however, to clarify the scope of the paradigm claim and the current state of empirical evidence. The integrated architecture proposed here is intended for a specific class of bioinformatics problems, those characterized by heterogeneous data modalities, sparse datasets, and regulated decision contexts, rather than as a universal replacement for all computational workflows. Simpler tasks such as protocol automation or literature synthesis may be adequately addressed with fewer components.

Furthermore, no currently published system has yet achieved the complete integration of foundation models, multi-agent orchestration, and automated validation within a prospectively validated clinical or drug discovery pipeline. Existing implementations typically address only subsets of this architecture. The SMA workflow discussed in this review should therefore be interpreted as an operational blueprint rather than a completed implementation.

Beyond these architectural considerations, several operational challenges remain central to the safe deployment of agentic systems in biomedical environments.

Interpretability must be treated as an operational constraint rather than a *post hoc* explanatory feature. In drug discovery settings, prioritization of targets, compounds, or safety signals must be defensible to multidisciplinary teams and regulatory reviewers. In clinical contexts, recommendations must align with established guidelines and institutional policies.

Bias, representativeness, and distributional shift constitute additional structural challenges. Foundation models and agent frameworks inevitably inherit biases present in the scientific literature, curated databases, and institutional health records. Underrepresented populations and rare diseases are frequently sparsely documented, creating blind spots that may lead to unsafe generalization when systems are deployed across heterogeneous clinical environments [49].

Technical integration with real-world clinical infrastructure often represents the most immediate barrier to deployment. Electronic health record systems, laboratory information systems, and medical devices operate under strict authentication, access control, and latency constraints. Robust deployment therefore requires not only model validation but also full lifecycle engineering, including rollback mechanisms, observability dashboards, and governance protocols.

Regulatory and ethical frameworks must evolve in parallel with technological capability. Agentic systems blur the boundary between decision support and semi-autonomous action, raising questions regarding accountability, informed consent, liability, and post-market surveillance.

**Figure 3:**
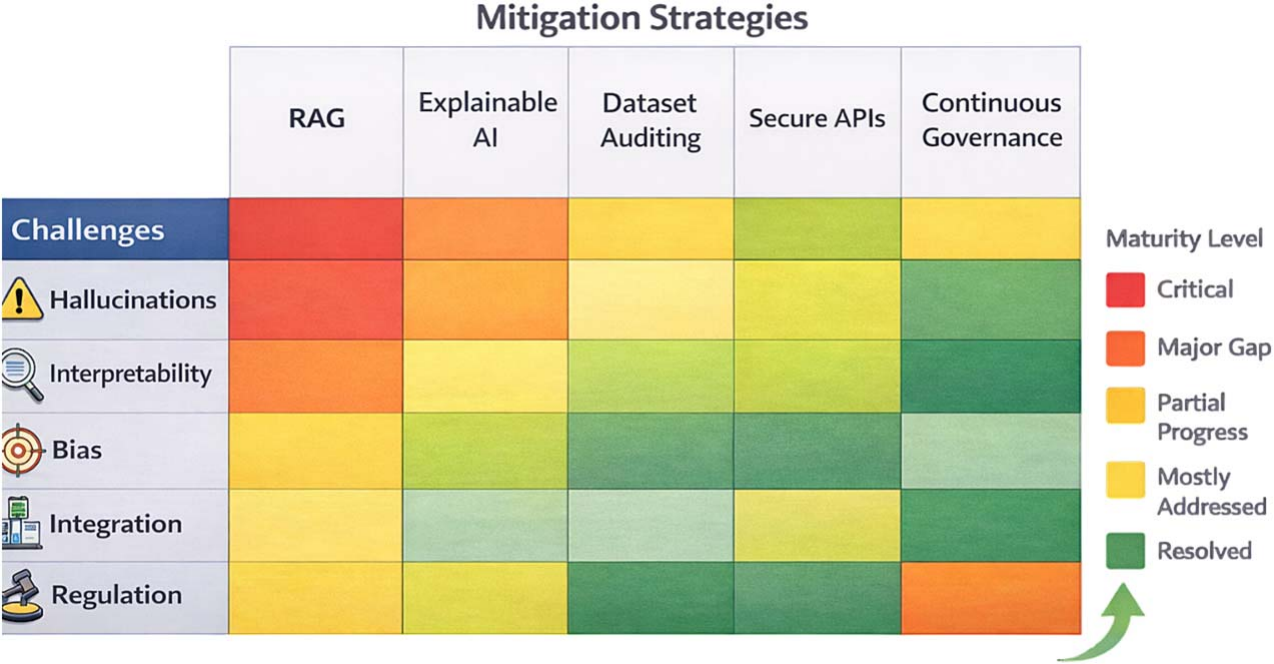
Challenges versus mitigation strategies heatmap. Rows represent the five main challenges discussed in the text (hallucinations, interpretability, bias, clinical integration, regulatory compliance); columns represent five mitigation families (RAG, explainable AI, dataset auditing, secure APIs, continuous governance). Shading intensity indicates the current maturity level of each solution in addressing each challenge, based on literature and current implementations reviewed in this paper. The scale ranges from low maturity (critical gap, indicated by the lightest/least intense shading) to high maturity (resolved, indicated by the darkest/most intense shading). The heatmap provides an at-a-glance assessment of the field’s status and highlights priority areas for future research. For instance, while RAG shows high maturity for addressing hallucinations, its maturity for interpretability remains low; conversely, continuous governance frameworks show low maturity across all dimensions, underscoring the need for parallel technical and regulatory innovation.

To synthesize this complex landscape of challenges and mitigation strategies, Fig. 3 presents a heatmap that cross-references the five main obstacles discussed in this section, i.e. hallucinations, interpretability, bias, clinical integration, and regulatory compliance, with five families of potential solutions.

Looking forward, several research and engineering directions appear particularly promising. Continual learning and deployment-time adaptation may allow agents to remain aligned with evolving biomedical literature and clinical standards. Federated and privacy-preserving adaptation offers a pathway to improving representativeness without centralizing sensitive patient data.

Finally, the concept of an “AI Agent Hospital” may serve as a strategic north star for the field. Such a system would not represent a fully autonomous clinical entity but rather a governance-aware, interoperable layer of coordinated agents operating across clinical, laboratory, and research infrastructures.

**Key Points**

- Agentic AI represents the next paradigm in bioinformatics**,** moving beyond prediction and generation to systems that plan, act, reflect, and iterate, enabling end-to-end scientific discovery through multi-agent coordination and foundation models.
- Universal biological representations (scGPT, Nicheformer, and EpiAgent) and multi-agent architectures (planning, action, reflection, and memory) together form the technical foundation for applications in drug discovery, personalized medicine, and laboratory automation.
- Realizing this vision requires addressing critical challenges**,** i.e. hallucinations, interpretability, bias, clinical integration, and regulatory gaps, through robust validation, retrieval-augmented generation, and continuous governance frameworks that ensure reliability and reproducibility.

## Data Availability

No datasets have been utilized in this review paper.

## Abbreviations

AI, Artificial Intelligence; API, Application Programming Interface; ATAC-seq, Assay for Transposase-Accessible Chromatin using sequencing; cCRE, candidate cis-Regulatory Elements; DNA, Deoxyribonucleic Acid; EHR, Electronic Health Record; LLM, Large Language Model; qPCR, Quantitative Polymerase Chain Reaction; RAG, Retrieval-Augmented Generation; RNA-seq, RNA sequencing; scRNA-seq, single-cell RNA sequencing; SMA, Spinal Muscular Atrophy.
